# Pharmacogenomics of human P450 oxidoreductase

**DOI:** 10.3389/fphar.2014.00103

**Published:** 2014-05-09

**Authors:** Amit V. Pandey, Patrick Sproll

**Affiliations:** ^1^Division of Pediatric Endocrinology, Department of Pediatrics, University Children's Hospital BernBern, Switzerland; ^2^Program in Molecular Life Sciences, Department of Biology, University of BernBern, Switzerland

**Keywords:** P450 oxidoreductase, P450 oxidoreductase deficiency, Antley–Bixler syndrome, steroid metabolism, disorders of sexual development, drug metabolism, P450c17, aromatase

## Abstract

Cytochrome P450 oxidoreductase (POR) supports reactions of microsomal cytochrome P450 which metabolize drugs and steroid hormones. Mutations in POR cause disorders of sexual development. P450 oxidoreductase deficiency (PORD) was initially identified in patients with Antley–Bixler syndrome (ABS) but now it has been established as a separate disorder of sexual development (DSD). Here we are summarizing the work on variations in POR related to metabolism of drugs and xenobiotics. We have compiled mutation data on reported cases of PORD from clinical studies. Mutations found in patients with defective steroid profiles impact metabolism of steroid hormones as well as drugs. Some trends are emerging that establish certain founder mutations in distinct populations, with Japanese (R457H), Caucasian (A287P), and Turkish (399–401) populations showing repeated findings of similar mutations. Most other mutations are found as single occurrences. A large number of different variants in POR gene with more than 130 amino acid changes are now listed in databases. Among the polymorphisms, the A503V is found in about 30% of all alleles but there are some differences across different population groups.

## Introduction

**Figure 1:**
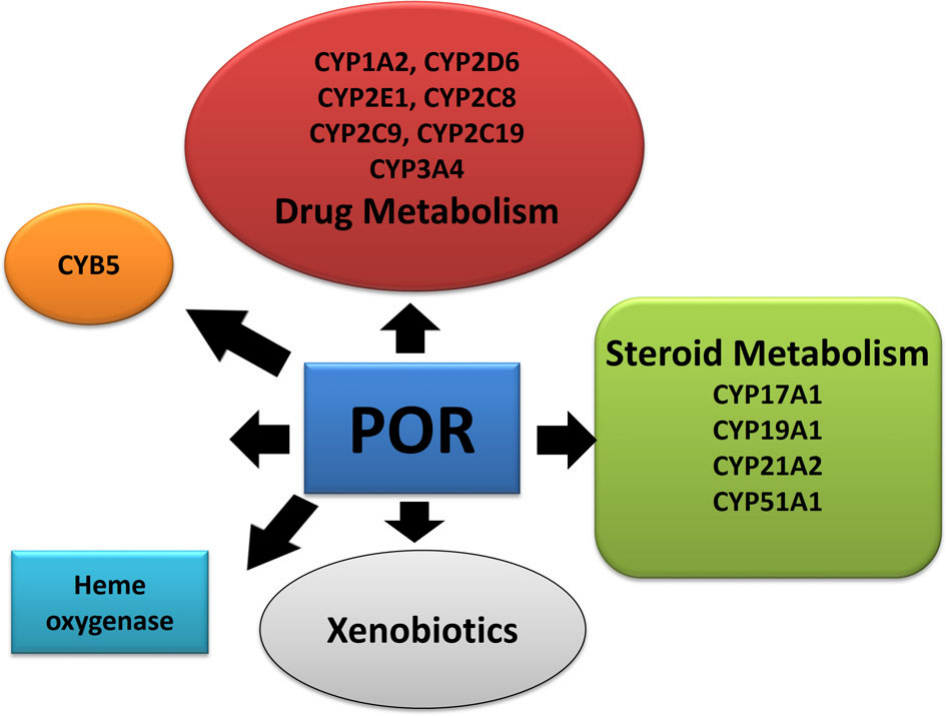
**Role of P450 oxidoreductase in biochemical pathways**. POR is required for multiple metabolic processes especially the microsomal P450 enzymes involved in metabolism of xenobiotics.

**Figure 2:**
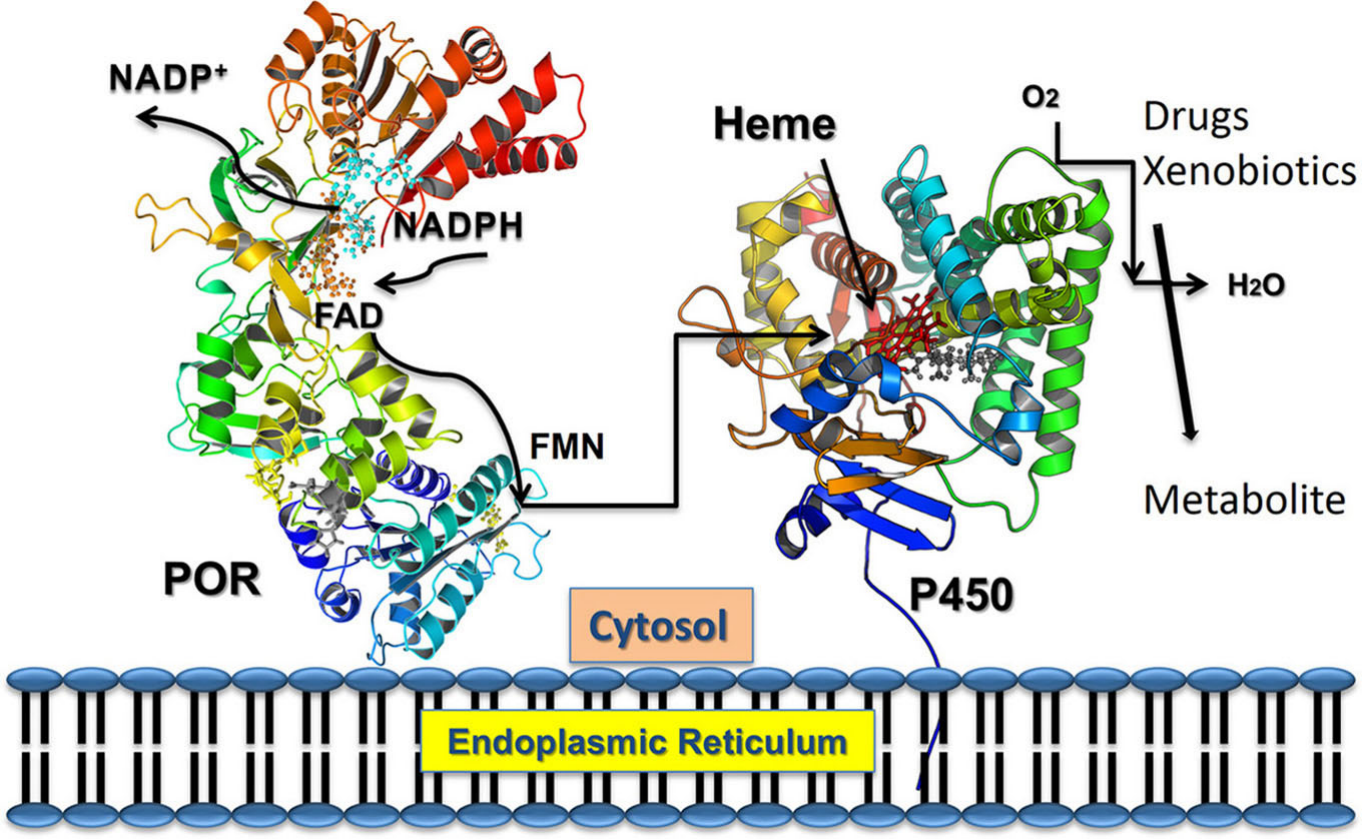
**Electron transfer from NADPH to redox partners of POR**. NADPH binds to POR located into the endoplasmic reticulum, and donates electrons which are received by FAD. Electron transfer to FAD causes a conformational change that brings together the FAD and FMN domains and electrons are transferred from FAD to FMN. The FMN domain of POR interacts with the P450s and other redox partners and completes the final step of electron transfer.

Cytochrome P450 reductase (POR) has a major role in metabolism of drugs and steroids (Flück et al., 2007; Pandey and Flück, 2013; Riddick et al., 2013) (Figure 1). All microsomal cytochrome P450 get electrons from nicotinamide adenine dinucleotide phosphate (NADPH) through POR (Zanger and Schwab, 2013) (Figure 2). A single gene of 71,754 bp located on chromosome 7 (locus 7q11.23) encodes human POR (Table 1). The human POR gene has one non-coding exon and 15 protein-coding exons and encodes a 680 amino acid membrane bound protein (RefSeq protein: NP_000932, UniProt P16435). Disruption of POR affects all microsomal P450 enzyme activities (Flück et al., 2007; Pandey and Flück, 2013). POR also supplies electrons to many other interaction partners. POR knockout mice are embryonic lethal (Shen et al., 2002; Otto et al., 2003). The liver-specific knockout of POR produces mice with lower metabolism and lipid accumulation (Gu et al., 2003; Henderson et al., 2003; Porter et al., 2011).

**Table 1:** **Database accession numbers for human POR**.

RefSeq Protein	NP_000932
RefSeq RNA	NM_000941
UniGene	Hs.354056
Uniprot	P16435
GeneID	5447
GeneCards	GC07P075528
Ensembl	ENSG00000127948
HGNC	HGNC:9208

**Figure 3:**
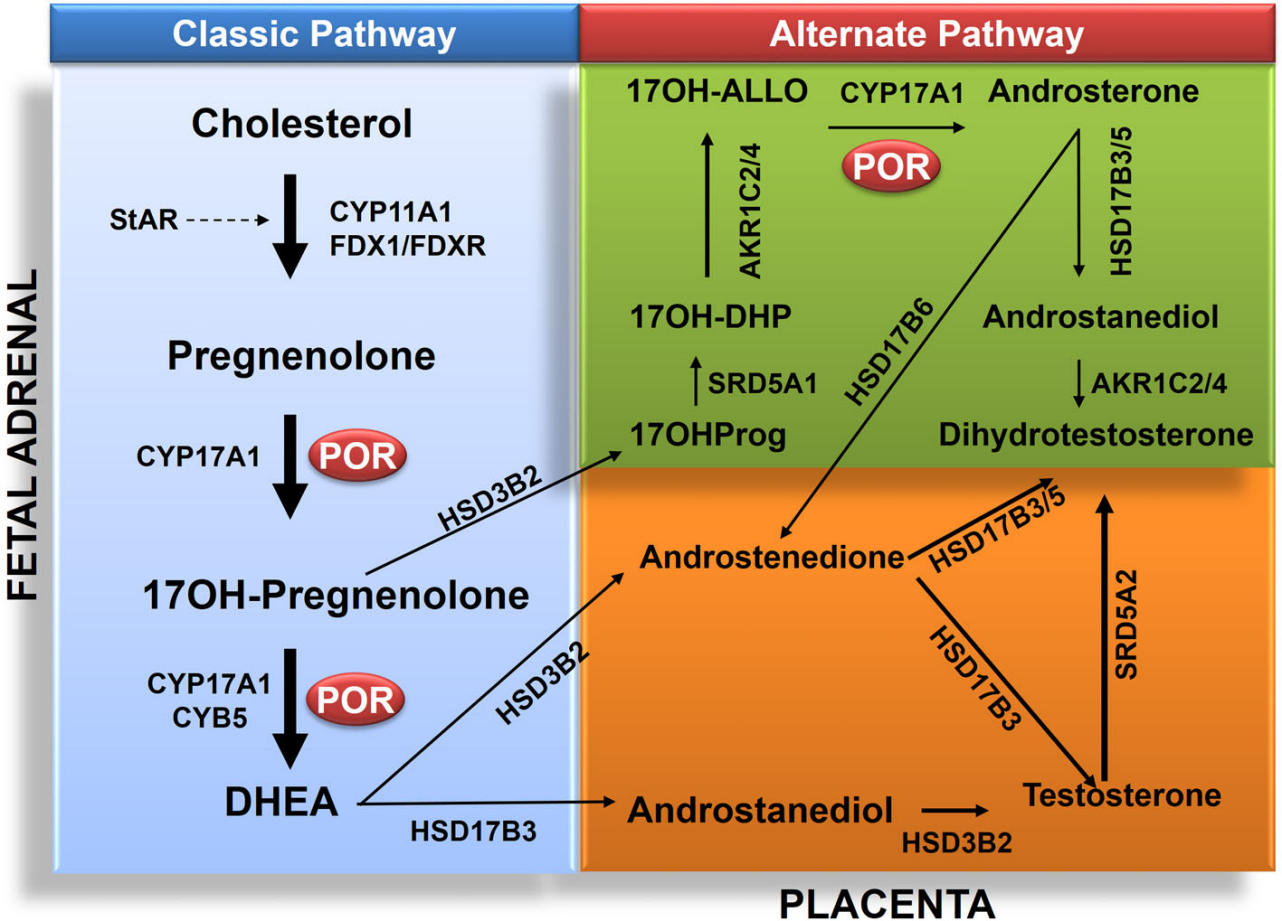
**Role of POR in biosynthesis of androgens in classic and alternate pathways**. The classic pathway proceeds from transfer cholesterol to mitochondrion through steroidogenic acute regulatory protein (StAR). In the mitochondrion cholesterol is converted to pregnenolone by CYP11A1. The biosynthesis process then continues from pregnenolone to 17OHPreg and DHEA to androstenedione or androstenediol and then to testosterone in testicular Leydig cells. Testosterone (T) is converted to dihydrotesterone (DHT) in genital skin. The backdoor pathway proceeds from 17OHPreg to 17OHProg, 17OH-DHP, 17OH-Allo, androsterone, androstanediol (A'diol) and then to DHT in the testis. Abbreviations: CYP11A1, P450scc, cholesterol side-chain cleavage enzyme; StAR, steroidogenic acute regulatory protein; FDX1, Adrenodoxin; FDXR, NADPH adrenodoxin oxidoreductase; CYP17A1, P450c17, 17α-hydroxylase/17,20-lyase; HSD3B2, 3β HSD2, 3β-hydroxysteroid dehydrogenase, type 2; CYB5, cytochrome b_5_; POR, P450 oxidoreductase; HSD17B3, 17β HSD3, 17β-hydroxysteroid dehydrogenase, type 3; and SRD5A2, 5α-reductase, type 2. The alternative pathway has four additional enzymes: SRD5A1, 5α-reductase, type 1; AKR1C2, Aldo-keto reductase 1C2, 3αHSD3; and AKR1C4, Aldo-keto reductase 1C4, 3αHSD1, for reductive 3αHSD activity; and HSD17B6, 17β HSD6, 17β-hydroxysteroid dehydrogenase, type 6; and/or AKR1C2/4 for oxidative 3αHSD activity. Full steroid names: 17OHPreg, 17-hydroxypregnenolone; 17OHProg, 17-hydroxyprogesterone; 17OH-DHP, 17-hydroxydihydroprogesterone (5α-pregnan-3α,17α-ol-20-one); 17OH-Allo, 17-hydroxyallopregnanolone (5α-pregnan-3α,17α-diol-20-one; P'diol); A'diol, androstanediol; T, testosterone; DHT, dihydrotestosterone, DHEA, dehydroepiandrosterone.

**Figure 4:**
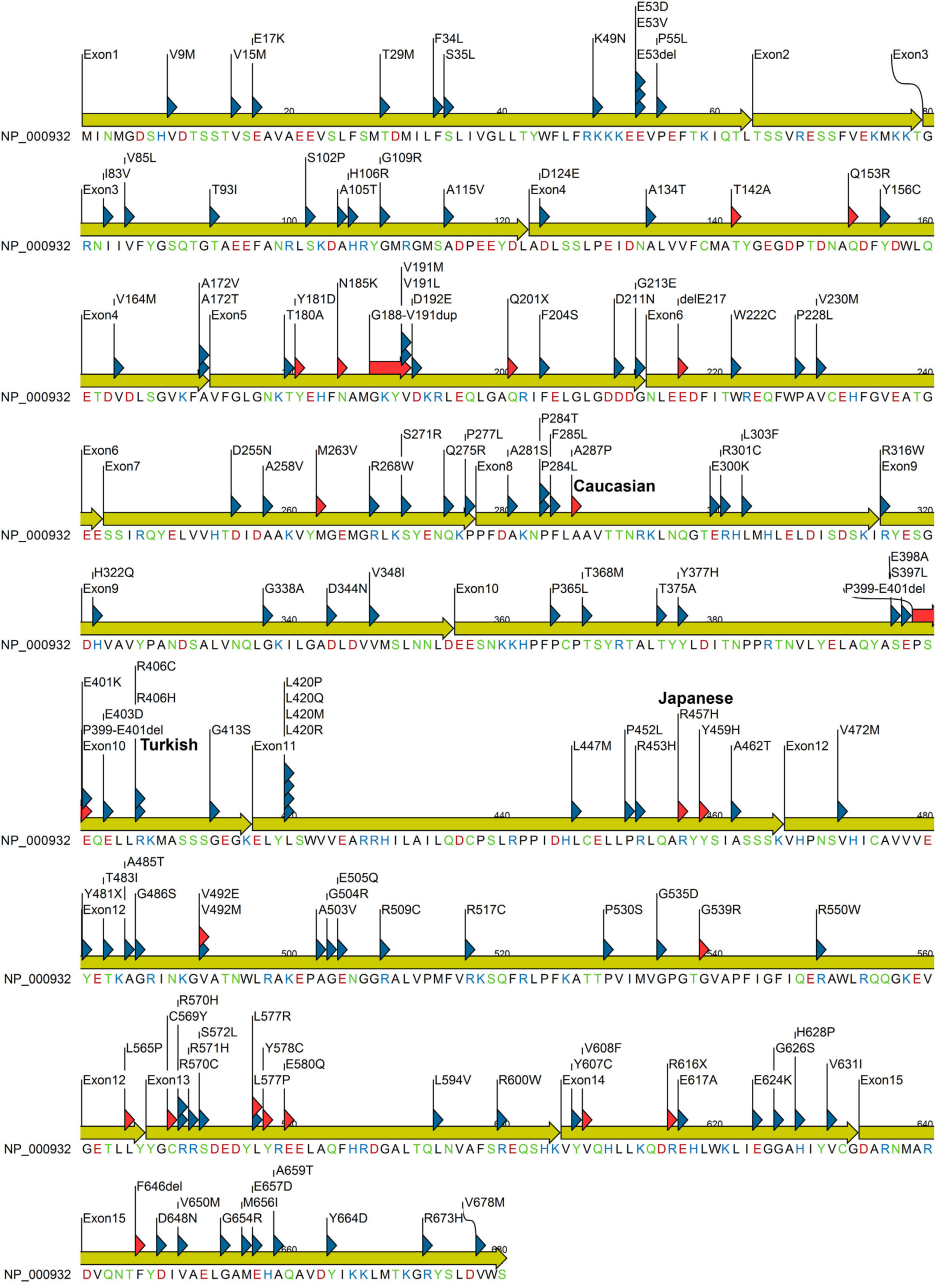
**POR protein sequence showing missense mutations identified in patients and variations from normal human population**. Mutation found in patients are indicated with red triangle while variants identified from healthy individuals are indicated with blue triangle. Amino acids are colored based on chemical properties; acidic residues (D, E) are in red, basic residues (H, R, K) are in blue, and rest of the polar amino acids (N, C, Q, S, T, and Y) are in green. Data were compiled from information available in NCBI SNP database and POR listing at CYPallele database (Sim et al., 2009) as of April 2014.

Cytochrome P450 oxidoreductase deficiency (PORD); (OMIM: 613571 and OMIM: 201750) is a form of congenital adrenal hyperplasia (Flück et al., 2004; Miller et al., 2004; Pandey et al., 2004; Flück and Pandey, 2013). Clinical symptoms of PORD were described in a 46,XY patient with disorder of sexual development (DSD) (Peterson et al., 1985). Sequencing of the POR gene in a Japanese 46,XX girl with a metabolic profile of steroid deficiency and symptoms of Antley–Bixler Syndrome (ABS) led to the characterization of PORD (Flück et al., 2004). Skeletal malformation in many ABS patients with ambiguous genitalia and defective steroid metabolism have now been linked to PORD (Huang et al., 2005). PORD is now listed as a separate metabolic disorder (Huang et al., 2005). An overview of reported cases shows that PORD disrupts steroid biosynthesis in adrenal gland and gonads (Huang et al., 2005; Dhir et al., 2007; Pandey et al., 2007; Flück et al., 2011a). This often leads to genital ambiguity at birth in both male and female. The absence of testosterone (T)/dihydrotestosterone (DHT) leads to development of female external genitalia (Flück and Pandey, in press). The presence of T/DHT triggers the formation of male genitalia. In humans the testosterone is mainly produced in testes by a process that starts by the conversion of pregnenolone to 17-hydroxypregnenolone (17-OHPreg) and dehydroepiandrosterone (DHEA) by the enzyme CYP17A1 in the adrenal (Miller and Auchus, 2011) (Figure 3). Like other microsomal P450 CYP17A1 requires POR (Miller and Auchus, 2011; Pandey and Flück, 2013). Aromatase converts androgens to estrogens and also requires POR (Pandey et al., 2007; Flück et al., 2011a; Bouchoucha et al., 2014). Thus, a defective POR affects sexual development (Flück and Pandey, 2011; Fukami et al., 2013) in both male and female. Many cases still do not have final diagnosis and other genes may be involved (Flück and Pandey, in press). Recently we have reported the first human mutations in aldo-keto reductases AKR1C2 (OMIM: 614279) and AKR1C4 (OMIM: 600451) (Flück et al., 2011b). This established two distinct pathways for the biosynthesis of androgens in fetal testis for control of sexual differentiation between male and female (Flück et al., 2011b; Biason-Lauber et al., 2013a,b; Flück and Pandey, in press). POR also plays a role in this alternate pathway (Biason-Lauber et al., 2013a; Pandey and Flück, 2013) (Figure 3). A large number of POR variants containing more than 130 amino acid changes in the POR protein have now been reported (Figure 4) by multiple researchers, and a list of variants in human POR is available at CYP allele website (www.cypalleles.ki.se/por.htm) (Huang et al., 2008; Sim et al., 2009; Flück et al., 2011a; Tomkova et al., 2012).

This review describes current status of information and research on disease related as well as naturally occurring variants of the POR gene. We have collected data on reports of PORD to summarize the current information on effects of POR mutations on enzymatic activities of redox partners. In addition, we have compiled the list of naturally occurring variants in POR gene that have been reported by researchers specifically sequencing the POR gene in distinct populations. We have also included information on POR variants available in genomic databases from large scale sequencing studies. Recently many POR variants have been studied for effects on drug metabolizing enzymes. We have also summarized studies describing the impact of individual POR variants on *in vitro* as well as *in vivo* drug metabolism cytochrome P450 activities. We have labeled the POR variants found in patients with disorders of steroid metabolism as “mutants” and variants identified from normal human population as “polymorphisms” (Figure 4). List of POR variants was compiled from publication of PORD case reports, POR sequencing studies, and human genome sequencing projects available through NCBI databases (http://www.ncbi.nlm.nih.gov).

## POR and steroid metabolism

**Figure 5:**
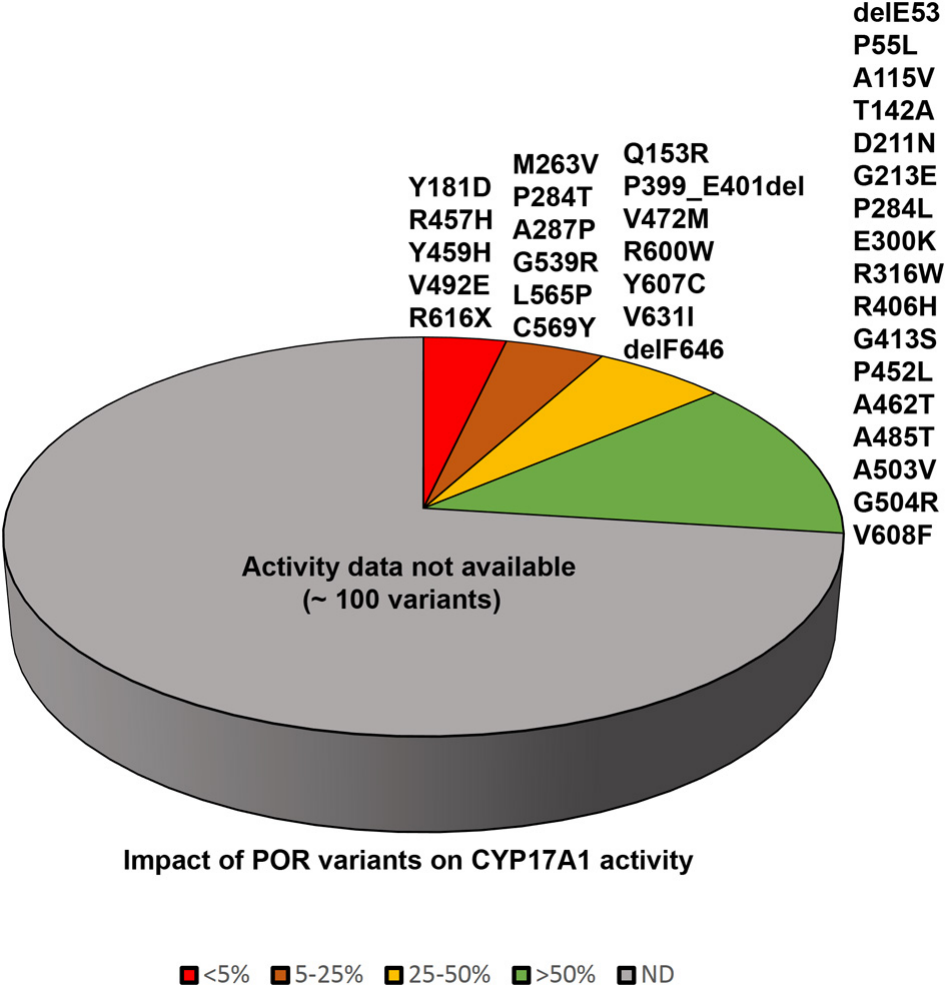
**Impact of POR variants on CYP17A1 activity**. A pie chart showing the impact of POR variants on CYP17A1 17,20 lyase activity. The variants which had <5% of WT activity are grouped as red, variants with 5–25% of WT activity are in orange, variants with 25–50% of activity are in yellow, and variants with >50% activity are in green. A large number of recently identified variants from large scale sequencing studies have not been tested in enzymatic assays and these are grouped in gray color pie.

PORD was identified as a DSD resulting from disruption of steroid metabolizing cytochrome P450 activities (Flück et al., 2004; Pandey et al., 2004). After the identification of POR variants in normal human population, early reports analyzed the impact of POR variants on three microsomal cytochrome P450 involved in steroid metabolism (CYP17A1, CYP19A1, and CYP21A2) (Flück et al., 2004; Huang et al., 2005; Dhir et al., 2007; Pandey et al., 2007). A large number of POR mutations or polymorphisms (Huang et al., 2005; Flück et al., 2011a) have been studied and analyzed based on known structures of POR (Xia et al., 2011; Pandey and Flück, 2013). Many mutations in POR found in patients are in co-factor binding sites and cause severe POR deficiency. The p.A115V variation first found in patients seems to be a polymorphism (Huang et al., 2008) and shows similar to wild type activity in CYP17A1 assays. The p.T142A mutation is close to FMN binding site but shows more than 50% of WT activity (Huang et al., 2005). The mutation p.Q153R also near the FMN binding site, had reduced POR activity in CYP17A1 assays (Table 2 and Figure 5) (Huang et al., 2005). An XY patient of Canadian origin with symptoms of 17-, 21-hydroxylase had POR mutation p.G188_V191dup but enzyme activity data are not available (Krone et al., 2012). The mutation p.A287P is most common in patients from Europe (Huang et al., 2005). It retains 20–40% of WT activity in CYP17A1 assays (Table 2) but had no effect on either CYP19A1 (Flück et al., 2004; Huang et al., 2005; Pandey et al., 2007) or CYP21A2 activity (Dhir et al., 2007). The polymorphism p.R316W shows normal activity in most assays (Huang et al., 2005; Pandey and Flück, 2013). The mutation p.P399_E401del found in two unrelated Turkish families (Flück et al., 2011a) had 68–85% of WT activity in different P450 assays (Flück et al., 2011a). Polymorphism p.G413S was identified in a sequencing study by Bioventures Inc. (Solus et al., 2004), retains wild type activity in many assays (Table 3) (Huang et al., 2005). A DSD patient of Pakistani origin (17 and 21-hydroxylase deficiencies) had p.R498P mutation (Krone et al., 2012). No enzymatic data for p.R498P are available. The variations p.A503V and the p.G504R have >50% activity (Huang et al., 2005). A link between p.A503V variant of POR and altered steroid profile in normal population was not found (Utriainen et al., 2012). The mutation p.G539R had <10% activity in the 17,20 lyase assay and 46% activity in the 17α-hydroxylase assay (Huang et al., 2005). The mutation p.C569Y in human POR (Flück et al., 2004) had 13% of 17,20 lyase activity and 28% of 17α-hydroxylase activity (Huang et al., 2005). The polymorphism p.R600W has 7–60% of WT activity in different assays (Huang et al., 2008). The p.C569Y mutation was compound heterozygote together with p.V608F (Flück et al., 2004). The p.V608F retained 57% of 17,20 lyase activity and 80% of 17α-hydroxylase activity (Table 2 and Figure 5) (Huang et al., 2005; Pandey et al., 2007). Both p.C569Y and p.V608F had more severe effects on CYP19A1 activity than on CYP17A1 activities (Flück et al., 2004; Huang et al., 2005; Pandey et al., 2007). The p.H628P affected both CYP17A1 and CYP21A2 activities to similar extent (Dhir et al., 2007). The p.Y607C had greater effects on CYP17A1 and CYP19A1 activities but CYP21A2 activity was 80% of WT (Idkowiak et al., 2010). There were no severe malformations in the p.Y607C patient (Huang et al., 2005; Idkowiak et al., 2010). A normal steroid profile also suggests normal activities of steroidogenic enzymes *in vivo* (Idkowiak et al., 2010). The p.V631I retained 40–50% of activity in the CYP17A1 assays. Deletion of F646 had no effect on 17α-hydroxylase activity but caused a 54% reduction in 17,20 lyase activity (Huang et al., 2005). Other reported mutants in the region p.Y578C and p.E580Q were not tested in enzymatic assays (Pandey and Flück, 2013).

**Table 2:** **Influence of POR variants found in patients with disorder of sexual development, on the enzyme activities of its redox partners**.

**POR**	**DBSNP id**	**CYP3A4**	**HO-1**	**Cyt c red**	**NADPH Oxi**	**CYP17A1 17OH-ase**	**CYP17A1 17,20 lyase**	**CYP19A1**	**CYP1A2**	**CYP2C19**	**CYP2D6**
WT	–	100	100	100	100	100	100	100	100	100	100
p.T142A	–	85	81	49	52	60	54	–	3	–	
p.Q153R	–	119	72	9	11	31	27	–	144	284	128
**p.Y181D**	rs72552771	–	–	–	–	–	–		–	–	
**p.N185K**				6	1	–	–				
p.M263V	–			76	57	9	13		92	136	
p.A287P	rs121912974	26	49	93	104	40	21	104	–	–	–
p.P339_E401del	–			20		26	31		30		
**p.R457H**	rs28931608	–	–	1	–	3	–	1	–	–	–
**p.Y459H**	–	–	–	–	–	11	–	–	–	–	
**p.V492E**	rs28931606	–	–	–	–	3	–	<1	–	–	
p.G539R	rs121912976			9	–	46	8		10	33	
p.L565P	–			14	1	32	19		–	–	
p.C569Y	rs28931607	32	33	18	7	28	13	51	6	–	
p.L577R				5	4	46	27				
p.Y607C	rs72557954			–	20	76	40		7	24	
p.V608F	rs72552772	16	32	8	3	80	57	24	5	–	
**p.R616X**	–	–	–	–	6	–	–	–	–	–	
p.V631I	rs145782750	89	96	74	23	51	40	–	6	23	
p.delF646	–	88	95	36	94	97	46	–	–	–	

*Activities are shown as percentage of the wild-type POR. POR, P450 oxidoreductase. Data were compiled from previous studies (Flück et al., 2004, 2010, 2011a; Pandey et al., 2004, 2007, 2010; Huang et al., 2005, 2008; Agrawal et al., 2008, 2010; Nicolo et al., 2010; Sandee et al., 2010). The V631I and Y607C were initially found in patients but later studies established those as normal population variants. Mutations listed in bold abolish all activity. These were found in patients with severe forms of disease and affected residues are located near co-factor binding sites of POR*.

*Enzymes/reactions and substrates (in parenthesis) listed in this table: CYP3A4: [BOMCC (7-Benzyloxy-4-trifluoromethylcoumarin)]; HO-1: Heme oxygenase-1 (heme); Cyt c red: Cytochrome c reduction (cytochrome c); NADPH ox: NADPH oxidation (NADPH); CYP17A1 17OH-ase: (Progesterone); CYP17A1 17,20 Lyase: (17-hydroxy Pregnenolone), CYP19A1: (androstenedione); CYP1A2, CYP2C19, and CYP2D6: [EOMCC (7-ethoxy-methyloxy-3-cyanocoumarin)]*.

**Table 3:** **Influence of POR variants found in normal population on the enzyme activities of its redox partners**.

**POR**	**DBSNP id**	**CYP3A4**	**HO-1**	**Cyt c red**	**NADPH Ox**	**CYP17A1 17OH-ase**	**CYP17A1 17,20 lyase**	**CYP19A1**	**CYP1A2**	**CYP2C19**	**CYP2D6**
WT	–	100	100	100	100	100	100	100	100	100	100
p.delE53	rs72553987			57	93	73	82		156	91	
p.P55L	rs72553988			67	123	82	115		114	83	
p.A115V	rs199634961	85	95	63	41	80	71	–	–	–	
p.D211N	rs72557914			27	59	57	62		52	52	
p.G213E	rs72557915			111	105	57	93		174	101	
p.P228L	rs17853284	101	106	75	72	100	41	–	20	39	
p.P284T	rs72557937			16	32	14	19		–	–	
p.P284L	rs72557938			104	153	44	82		41	27	
p.E300K	rs11540674			93	104	112	146		85		
p.R316W	BV12016#	110	96	61	77	94	141	–	109	81	
p.R406H	rs72557929			62	78	61	57		125	155	
p.G413S	BV12020#	100	104	76	100	83	110	–	73	104	
p.P452L	rs72557935			16	12	48	61		21	83	
p.A462T	rs72557936			85	69	58	66		119	100	
p.V472M	rs72557946			23	24	58	46		54	77	
p.A485T	rs72557947			36	51	68	88		80	98	
p.A503V	rs1057868	107	97	67	56	68	58	–	85	113	85
p.G504R	BV12031#	93	107	53	47	91	103	–	82	140	–
p.R600W	rs72557950			67	56	38	40		36	60	
p.Y607C	rs72557954			–	20	76	40		7	24	
p.V631I	rs145782750	89	96	74	23	51	40	–	6	23	

*Activities are shown as percentage of the wild-type POR. POR, P450 oxidoreductase. Data were compiled from previous reports (Flück et al., 2004, 2010, 2011a; Pandey et al., 2004, 2007, 2010; Huang et al., 2005, 2008; Agrawal et al., 2008, 2010; Nicolo et al., 2010; Sandee et al., 2010). # DBSNP id not available, these were identified in a sequencing study by Bioventures Inc. (Solus et al., 2004) and are represented as Bioventure ID numbers BVxxxxx*.

*Enzymes/reactions and substrates (in parenthesis) listed in this table: CYP3A4: [BOMCC (7-Benzyloxy-4-trifluoromethylcoumarin)]; HO-1: Heme oxygenase-1 (heme); Cyt c red: Cytochrome c reduction (cytochrome c); NADPH ox: NADPH oxidation (NADPH); CYP17A1 17OH-ase: (Progesterone); CYP17A1 17,20 Lyase: (17-hydroxy Pregnenolone), CYP19A1: (androstenedione); CYP1A2, CYP2C19 and CYP2D6: [EOMCC (7-ethoxy-methyloxy-3-cyanocoumarin)]*.

## POR and drug metabolism

After the discovery of POR mutations in patients and identification several variants of POR in healthy individuals we suggested that POR variants may also impact drug metabolizing P450 enzymes since POR is necessary for activities of all microsomal P450 (Flück et al., 2004, 2007; Pandey et al., 2004; Huang et al., 2005). Variations in POR may affect many different redox partners. Many recent studies reported effects of POR variants on several drug metabolizing P450s (Agrawal et al., 2008, 2010; Hart et al., 2008; Kranendonk et al., 2008; Gomes et al., 2009; Miller et al., 2009; Oneda et al., 2009; Flück et al., 2010; Marohnic et al., 2010; Nicolo et al., 2010; Tomalik-Scharte et al., 2010) and other metabolic processes (Agrawal et al., 2010; Flück et al., 2010; Marohnic et al., 2010; Nicolo et al., 2010; Pandey et al., 2010; Sandee et al., 2010; Zhang et al., 2011). Here we are describing the reports on human studies, an overview of metabolic activities in POR mouse models by laboratories of Roland Wolf and Xin-Xin Ding has been reviewed earlier (Riddick et al., 2013).

**Figure 6:**
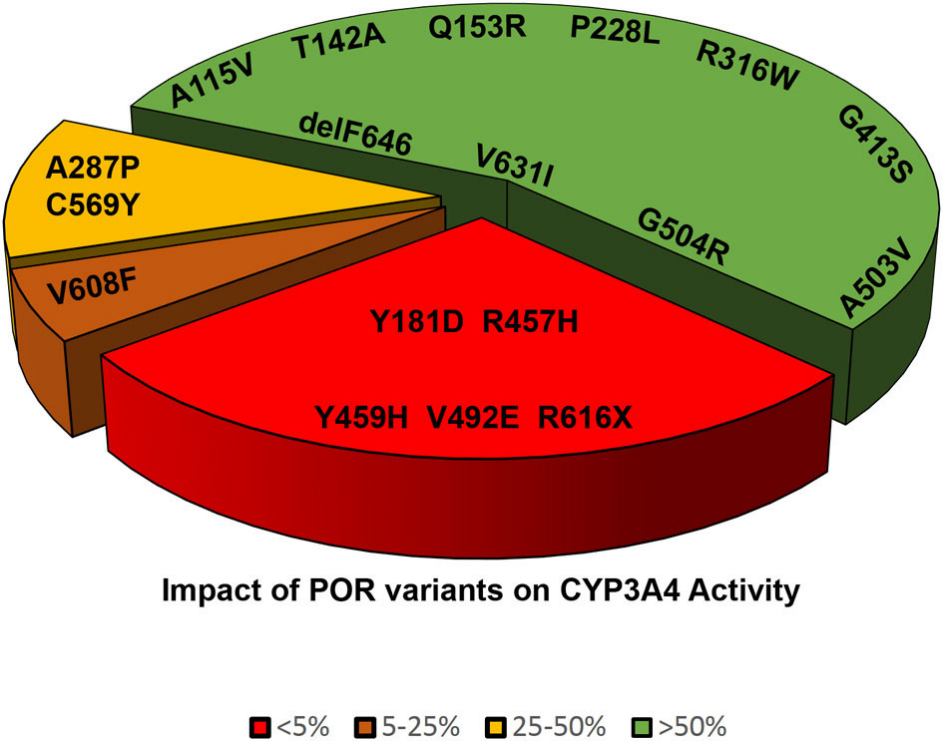
**Impact of POR variants on CYP3A4 activity**. A pie chart showing the impact of POR variants on CYP3A4 activity. The variants which had <5% of WT activity are grouped as red, variants with 5–25% of WT activity are in orange, variants with 25–50% of activity are in yellow, and variants with >50% activity are in green.

Cytochrome P450 3A4 (CYP3A4) metabolizes a wide range of drugs and xenobiotics (Klein and Zanger, 2013; Meyer et al., 2013; Zanger and Schwab, 2013). POR mutations affect CYP3A4 activities *in vitro* (Nicolo et al., 2010). The POR mutant p.A287P (common mutation found in patients from “European” population) showed 75% loss of CYP3A4 activity (Table 2 and Figure 6) (Flück et al., 2010; Nicolo et al., 2010). The p.R316W and p.G413S variants had close to WT activities (Flück et al., 2010). The mutations disrupting FAD binding (p.R457H, p.Y459H and p.V492E) resulted in complete loss of CYP3A4 activity (Flück et al., 2010). POR polymorphisms p.A503V and p.G504R showed close to normal activities (Table 3) (Flück et al., 2010). POR mutations p.C569Y and p.V608F resulted in 65–85% loss of CYP3A4 and p.R616X lost all activity (Table 2) (Flück et al., 2010). The POR mutant p.Y181D resulted in almost total loss of CYP3A4 activity, while variant p.Q153R was normal and variant p.T142A had 75–80% activity (Flück et al., 2010). A larger study of POR variants with CYP3A4 suggests POR may change activities of CYP3A4 in patients (Flück et al., 2010; Nicolo et al., 2010). There may be substrate specific effects of POR variants on CYP3A4 activities. Agrawal et al. (2010) found that POR variants p.A287P and p.R457H had lower activity with all substrates. The p.Q153R had 76–94% of WT activity with midazolam and erythromycin, but showed 129–150% activity with testosterone and quinidine (Agrawal et al., 2010). The polymorphism p.A503V had 20–40% reduction in CYP3A4 activity with testosterone and midazolam. With quinidine and erythromycin the p.A503V has no significant change in activities (Agrawal et al., 2010). Studies from us and others have reported normal CYP3A4 activity with POR p.G413S (Flück et al., 2010; Moutinho et al., 2012). POR polymorphisms p.P228L, p.R316W, p.G413S, p.A503V, and p.G504R have 40–100% activity in most assays (Table 3). The p.A115V had less than 40–60% of activity, and p.V631I had 23–76% of wild-type activity (Huang et al., 2005).

Agrawal et al. (2008) found POR mutations p.A287P and p.R457H resulted in almost complete loss of CYP1A2 and CYP2C19 activities (Table 2). The polymorphism p.A503V had 85% of WT activity with CYP1A2 and 113% of WT activity with CYP2C19 (Table 3). The p.Q153R, a disease-linked mutation increased activity of CYP1A2 to 144% and CYP2C19 activity to 284% of WT.

In addition to CYP3A4 the CYP2D6 is another major drug metabolizing enzyme (Zanger and Schwab, 2013). POR mutants p.A287P and p.R457H have no CYP2D6 activity with synthetic substrate EOMCC (Sandee et al., 2010). The p.A287P mutant retained 25% of WT activity with dextromethorphan and bufuralol as CYP2D6 substrates. The p.Q153R had increased activity with CYP2D6. The p.A503V resulted in activities of 85% with EOMCC, 62% with dextromethorphan, and 53% with bufuralol (Sandee et al., 2010).

There may also be differences in effects of POR variants due to presence of P450 isoforms but, this has not been explored in detail. Subramanian et al. (2012) studied the polymorphic forms of CYP2C9 (*CYP2C9.1*, *CYP2C9.2*, and *CYP2C9.3*) with POR variants. POR variants p.A287P and p.R457H resulted in reduced activities with all three variants of CYP2C9. The disease related variant p.R153Q had higher activities with all variants of CYP2C9 (Subramanian et al., 2012).

## POR variants in different genetic populations

A study on sequencing of POR gene in 842 healthy people (Huang et al., 2008) detected 140 SNPs, and 43 of those were in ≥1% of alleles. 32 variants were in protein coding regions, 2 in untranslated exon 1U, 94 in the introns and 12 in 5′ flanking DNA. Among the 32 variations in protein coding region, 13 new variants resulted in amino acid change. The new variants were p.delE53, p.P55L, p.D211N, p.G213E, p.P284L, p.P284T, p.R406H, p.P452L, p.A462T, p.V472M, p.A485T, p.R600W, and p.Y607C. The POR polymorphism p.A503V (rs1057868) (Huang et al., 2005), was present in about 25% of all alleles (Huang et al., 2008). Most of the polymorphic variants had >40% of WT activities in different *in vitro* assays.

Some researchers have attempted to sequence POR gene in human liver samples to study the impact of POR variant in enzymatic studies in liver microsomes. A study of POR sequence in human liver samples by Xiao-Bo Zhong added 3 novel amino acid variations (p.K49N, p.L420M, and p.L577P) (Hart et al., 2008). The POR variant p.L577P had reduced activities in many drug metabolizing P450 assays (Hart et al., 2008). Another study of POR variation analysis by Ulli Zanger in 150 human liver samples found 43 variations with 19 SNPs in exonic regions (Gomes et al., 2009). They also found two combinatorial alleles of (p.P228L + p.A503V and p.A503V + p.V631I) and similar to previous reports, the p.A503V allele was the most common variant (Gomes et al., 2009).

**Figure 7:**
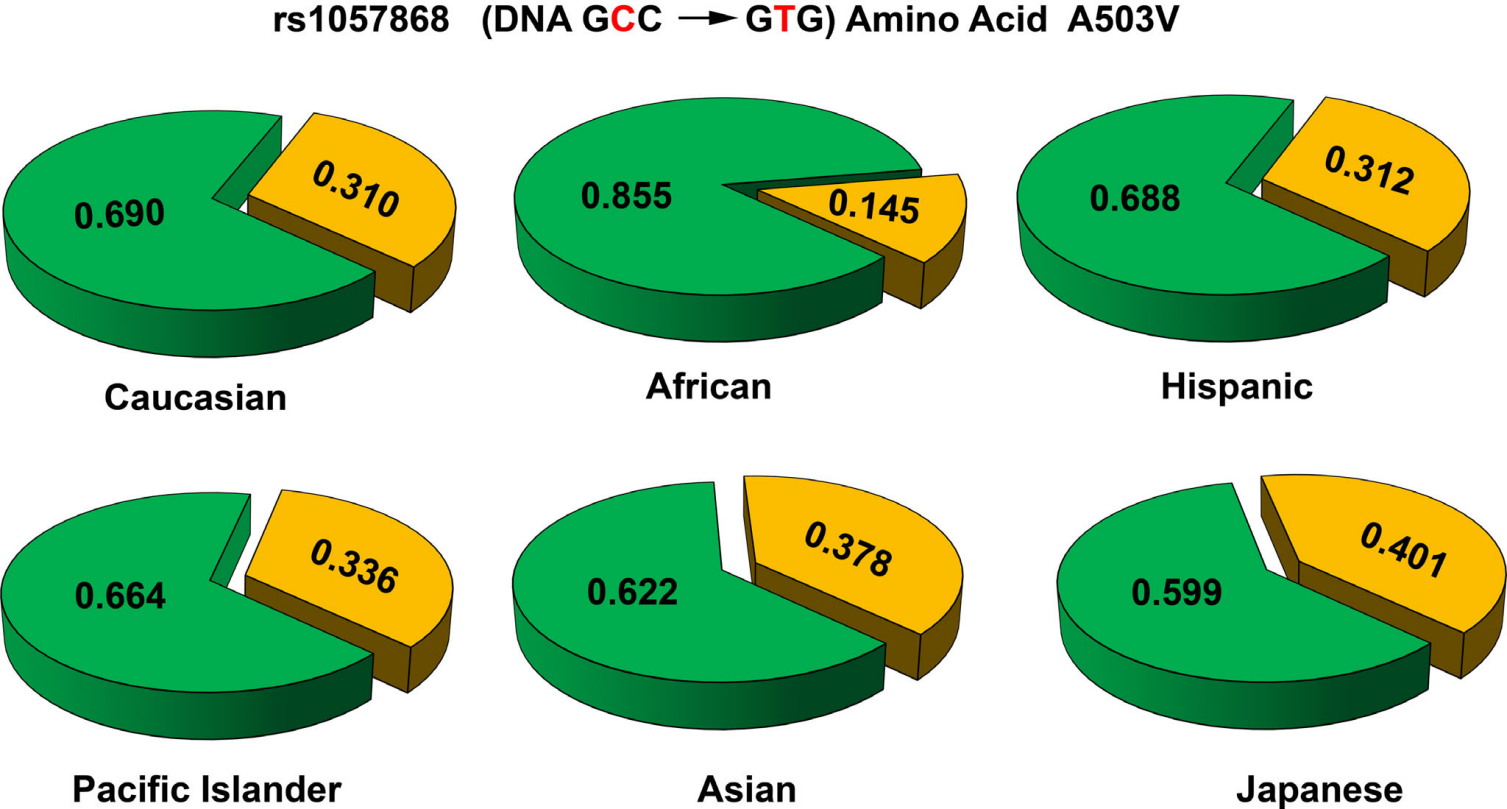
**Allele frequencies of POR variant A503V in different population groups**. The p.A503V variant of POR (rs1057868) is most common across all population groups. In various sequencing studies specifically targeting POR gene as well as large scale sequencing projects the POR p.A503V is present as most common minor allele. The average minor allele frequency in different studies ranges from 0.25 to 0.45. A closer look at the data revealed some differences. In the Caucasian and Hispanic populations the p.A503V allele is present in 31% of alleles, while in Pacific Islanders (34%) and Asian populations (38%) there is a slight but consistent increase. In the Japanese population the p.A503V frequency is highest (40%) while in African Americans it is present in less than 15% of all alleles. Alleles with p.503A are in green while p.503V is in orange. Data were compiled from information available in NCBI SNP database as of April 2014 (http://www.ncbi.nlm.nih.gov/SNP/snp_ref.cgi?rs=1057868).

**Figure 8:**
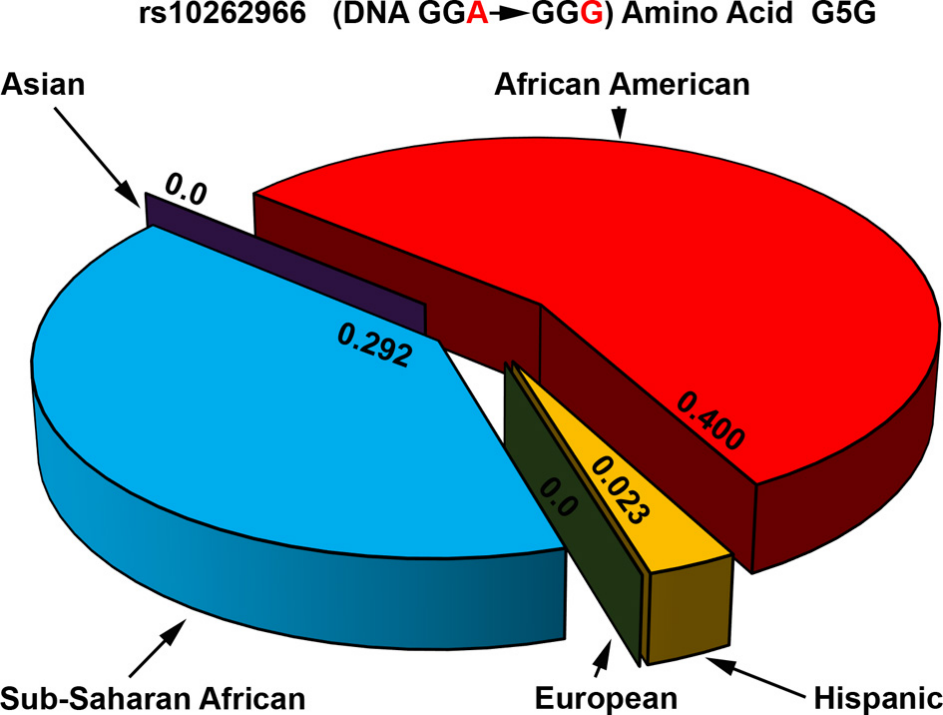
**Allele frequencies of POR variant G5G associated with increased breast cancer risk**. The p.G5G variant of POR (rs10262966) is associated with increased risk of breast cancer when present in homozygous form in African Americans (Haiman et al., 2007). Genomic data from various sequencing projects show a clear pattern for the presence of rs10262966 in different populations. In the Caucasian and Asian populations rs10262966 is not present, while in Hispanic population it is present with a minor allele frequency of 0.023. In the Sub-Saharan African population the rs10262966 frequency is higher at 0.297 while in African Americans it is present in 40% of all alleles. Data were compiled from information available in NCBI SNP database as of April 2014 (http://www.ncbi.nlm.nih.gov/SNP/snp_ref.cgi?rs=10262966).

Several new studies have sequenced POR gene in specific population groups. Sequencing of POR gene in 235 Japanese individuals (Saito et al., 2011) found 4 new amino acid changes (p.T29M, p.R550W, p.R570C, and p.A659T). Here p.A503V was present at a somewhat higher frequency of 0.434. A recent study sequenced POR variants in distinct Jewish populations and identified 6 novel amino acid variants, p.S102P, p.V164M, p.V191M, p.D344N, p.E398A, and p.D648N (Tomkova et al., 2012). The average minor allele frequency (MAF) of p.A503V in different reports have ranged from 0.25 to 0.45. Analysis of genomic sequence data available at NCBI SNP database shows that there are some differences among different genetic groups (Figure 7). In the Caucasian and Hispanic populations the p.A503V allele frequency is 31%, in Pacific Islanders 34%, and in Asian populations the p.A503V allele is present at 38%. In the Japanese population the p.A503V frequency is highest at 40% while the African Americans group has the lowest presence at less than 15% of all alleles. Homozygous presence of p.G5G (rs10262966, nucleotide change GGA to GGG) silent mutation has been associated with increased risk in African Americans (Haiman et al., 2007). This variant is not present in European and Asian populations and has a MAF of 0.023 in Hispanic population (Figure 8). In Sub-Saharan Africans it is present in 30% of alleles (MAF 0.297) and African Americans have the highest frequency with 40% of alleles (MAF 0.400) carrying the p.G5G variant of POR. The p.A50V variant was also tested for association with increased breast cancer risk in same study but no linkage was observed (Haiman et al., 2007).

## *In vivo* drug clearance studies examining the impact of POR variants

Several studies have now examined the role of POR variants on *in vivo* drug clearance using many different standard drugs as markers. One report has described *in vivo* drug metabolism activities in a POR p.A287P homozygous patient and her heterozygous mother (Tomalik-Scharte et al., 2010). Genotyping of P450 enzymes predicted normal to high P450 activities in both the patient and her mother. The *in vivo* results showed subnormal activities of CYP1A2, CYP2C9, CYP2D6, and CYP3A4 in the patient. The heterozygous mother had reduced CYP1A2 and CYP2C9 activities. These data are in agreement with disruption of *in vitro* CYP3A4 activities by p.A287P variant of POR reported by us (Flück et al., 2010; Nicolo et al., 2010). In patients with PORD, the *in vitro* and *in vivo* activities of POR dependent P450 may be taken into account to modify drug dosages and supplementation with steroids.

POR variant p.A503V (POR^*^28) had higher levels of CYP3A activities based on midazolam clearance (Oneda et al., 2009). Yang et al have studied the effect of POR variant p.A503V on *in vivo* CYP3A activity in healthy Chinese men (Yang et al., 2011). Out of 73 subjects (CC 21, TT11; CT 41) the midazolam metabolite ratio was greater in the TT group compared with carriers of the C allele. The p.A503V variant of POR had increased 1-hydroxylation of midazolam. Further analysis showed association with hepatic but not intestinal CYP3A activities (Yang et al., 2011). There was no significant differences in combined hepatic plus intestinal CYP3A activity.

A study tested the impact of POR p.A503V on metabolism of tacrolimus (de Jonge et al., 2011). They found lower levels of tacrolimus in carriers of p.A503V allele but in the individuals with CYP3A5^*^3/^*^3 allele, the p.A503V POR had no effect (de Jonge et al., 2011). Another study measured the impact of p.A503V (rs1057868, DNA change GCC to GTC) allele of POR on metabolism of tacrolimus in healthy Chinese men (Zhang et al., 2013). There was no difference in tacrolimus metabolism between two POR alleles. Further analysis showed no significant differences in tacrolimus pharmacokinetics in CYP3A5 non-expressers (CYP3A5^*^3/^*^3). The mean tacrolimus exposure for the p.A503V CC homozygotes in CYP3A5 expressers (CYP3A5^*^1/^*^1 or ^*^1/^*^3) were much higher than the p.A503V CT heterozygotes. These studies suggest a role for p.A503V allele of POR in the variability of tacrolimus exposure levels. *In vitro* studies of CYP3A5 activities comparing normal and p.A503V variants of POR may confirm this hypothesis.

A CYP1A2 genotyping study in smokers also checked POR polymorphic variants for potential linkage to metabolic defects (Dobrinas et al., 2012). During smoking there was no impact of any POR polymorphisms on CYP1A2 activity. CYP1A2 activity increased after smoking cessation in POR rs2302429A and rs1057868T (p.A503V) carriers. The carriers of POR rs2286823A and of rs17148944G-rs10239977C-rs3815455C-rs2286823A-rs2302429G-rs1057868C (503A) had decreased CYP1A2 activity.

## Conclusions

PORD is more complex than any single enzyme or protein deficiency (Janner et al., 2006; Flück et al., 2011c; Camats et al., 2012). It affects drug and xenobiotic metabolism pathways (Henderson et al., 2006; Flück et al., 2010; Nicolo et al., 2010). Variants of POR have different interactions with different redox partners. Effect of POR variants on drug metabolizing P450 enzymes needs detailed studies. Deposition of heme in liver lowers cytochrome P450 levels and enzymatic activities (Pandey et al., 1995, 1996). POR variants with lower activities may complicate these symptoms and result in a more severe disease. Heme oxygenase plays a major role in limiting the severity of malaria and sepsis infections (Pamplona et al., 2007; Larsen et al., 2010; Pandey et al., 2010; Ferreira et al., 2011). There may be potential links between the common POR variants and pathogenesis of infections. External flavin supplementation reverts loss of activity of some POR variants (Nicolo et al., 2010). An interesting possibility to explore in future experiments is whether external FMN can receive electrons from bound NADPH in POR as reported for a *B. subtilis* reductase (Deller et al., 2006). Whether flavin treatment may help patients with POR deficiency remains untested in clinical settings.

### Conflict of interest statement

The authors declare that the research was conducted in the absence of any commercial or financial relationships that could be construed as a potential conflict of interest.
